# All-cause mortality and hospital admissions for nursing home residents during the COVID-19 pandemic: a Norwegian register-based cohort study

**DOI:** 10.1186/s12916-024-03523-8

**Published:** 2024-08-07

**Authors:** Henning Øien, Jonas Gjesvik, Katrine Damgaard Skyrud, Torill Alise Rotevatn, Mari Grøsland

**Affiliations:** 1 Cluster of Health Services Research, Norwegian Institute of Public Health, Oslo, Norway; 2 Breast Cancer Screening, Norwegian Institute of Public Health, Oslo, Norway; 3 Department of Health Management and Health Economics, University of Oslo, Oslo, Norway

**Keywords:** Nursing home residents, Long-term care, COVID-19, Pandemic, Mortality, Hospital admissions

## Abstract

**Background:**

This paper investigates the consequences of the COVID-19 pandemic on mortality and hospitalization among nursing home residents in Norway. While existing evidence shows that nursing home residents were overrepresented among COVID-19-related deaths, suggesting inadequate protection measures, this study argues that the observed overrepresentation in mortality and hospitalization may partly stem from the inherent frailty of this demographic. Using nationwide administrative data, we assessed excess deaths and hospitalization by comparing pandemic-era rates to those of a pre-pandemic cohort.

**Methods:**

We compared mortality and hospitalization rates between a pandemic cohort of nursing home residents as of September 2019 (*N* = 30,052), and a pre-pandemic cohort as of September 2017 (*N* = 30,429). Both cohorts were followed monthly for two years, beginning in September 2019 and 2017, respectively. This analysis was conducted at the national level and separately for nursing home residents in areas with low, medium, and high SARS-CoV-2 community transmission. Event studies and difference-in-difference models allowed us to separate the impact of the pandemic on mortality and hospitalization from secular and seasonal changes.

**Results:**

The pandemic cohort experienced a non-significant 0.07 percentage points (95% confidence interval (CI): − 0.081 to 0.221) increase in all-cause mortality during the 18 months following pandemic onset, compared to the pre-pandemic cohort. Moreover, our findings indicate a substantial reduction in hospitalizations of 0.27 percentage points (95% CI: − 0.464 to − 0.135) and a non-significant decrease of 0.80 percentage points (95% CI: − 2.529 to 0.929) in the proportion of nursing home residents hospitalized before death. The effect on mortality remained consistent across regions with both high and low levels of SARS-CoV-2 community transmission.

**Conclusions:**

Our findings indicate no clear evidence of excess all-cause mortality in Norway during the pandemic, neither nationally nor in areas with high infection rates. This suggests that early implementation of nationwide and nursing home-specific infection control measures during the pandemic effectively protected nursing home residents. Furthermore, our results revealed a decrease in hospitalizations, both overall and prior to death, suggesting that nursing homes adhered to national guidelines promoting on-site treatment for residents.

**Supplementary Information:**

The online version contains supplementary material available at 10.1186/s12916-024-03523-8.

## Background

In March 2020, governments worldwide implemented stringent social distancing measures and stay-at-home orders in response to the spread of the SARS-CoV-2 virus that caused the COVID-19 pandemic. Nursing homes, housing frail older people, were no exception to these measures, aligning with recommendations from the World Health Organization [1]. Despite these efforts, the pandemic response in American and European nursing homes was criticized because nursing home residents were highly overrepresented among those who died of COVID-19 [2–4]. During the initial year of the pandemic, nursing home residents, constituting a small fraction of the population (less than 0.5% in the United States (US) and 1% in Europe), accounted for a disproportionately high share of COVID-19 deaths, reaching up to 25% in the US [2], and 31 to 80% of COVID-19 deaths in Europe [3].

Although there is clear evidence of structural problems in the pandemic response in US and European nursing homes [2, 4–7], it is expected that without extreme mitigation measures, elderly and frail nursing home residents would be disproportionately affected by respiratory diseases, including COVID-19. Frail older people are typically multimorbid, have weakened immune systems, and frequently exhibit atypical disease symptoms [8]. This makes it harder to detect COVID-19 among nursing home residents and to prevent infection entry and spread in nursing homes without imposing strict mitigation measures [9]. These factors plausibly contributed to the overrepresentation of nursing home residents among COVID-19 fatalities in countries with relatively high rates of community transmission (such as Sweden, the US, and the United Kingdom (UK) [10–13]) as well as in countries with relatively low rates (such as Canada, Denmark, and Norway [14, 15]). Since deaths and hospitalizations are common among nursing home residents, a better way to assess the impact of the COVID-19 pandemic on the nursing home population is to compare the rates of all-cause mortality and hospitalization of nursing home residents during the pandemic to residents in a pre-pandemic period [16].

There is some evidence of excess all-cause mortality and hospitalizations among nursing home residents. Studies in Sweden, Italy, England, Canada, and the US have reported higher all-cause mortality rates in long-term care facilities during the pandemic [10–12, 14, 17]. Additionally, evidence indicates a decrease in hospitalization rates among residents in skilled nursing facilities in the US during the pandemic [11, 18].

Learning how COVID-19 has affected nursing home residents in countries with varying transmission rates and mitigation strategies is important. This can help policymakers and public health authorities create targeted interventions to safeguard residents and prevent the spread of disease in future pandemics. This study aimed to contribute to our understanding of the impact of the COVID-19 pandemic on nursing home residents by comparing mortality and hospitalization rates in pre- and post-pandemic cohorts in Norway. To investigate the consequences of the pandemic, administrative data for all Norwegian nursing home residents as of September 2019 were analyzed, tracking their mortality rates and healthcare utilization from September 2019 to August 2021. Using difference-in-differences and event study models, these outcomes were compared to a pre-pandemic cohort of nursing home residents in September 2017. We further analyzed cause-specific hospitalizations to identify which conditions experienced shifts in admission rates during the pandemic. Given the significant variation in SARS-CoV-2 infection rates across different regions in Norway, we categorized nursing home residents based on the community transmission rates in their respective regions—low, medium, and high. We then examined excess mortality and hospitalization rates within each category to assess the impact of transmission intensity on these outcomes.

## Methods

### Institutional setting

In Norway, universal health coverage ensures that all residents have access to highly subsidized specialized and primary healthcare services. The 356 municipalities are responsible for primary care, nursing home care, and other long-term care services. Nursing homes are staffed with physicians, nurses, and other healthcare workers and offer 24-h health and care services for those who are unable to live independently [19, 20]. Over time, nursing home care has almost exclusively become a service for older people with severe physical or cognitive impairment. This is driven by the principle that people with functional disabilities should be given services at the “lowest level of efficient care” to be able to live at home as long as possible (20, 21).

### COVID-19 mitigation measures

During the pandemic, comprehensive national lockdowns were instituted for the entire population, including stay-at-home orders, limitations on visitor numbers, and social distancing restrictions, alongside rigorous testing, quarantine, and isolation procedures [21]. Throughout our pandemic study period, the strictest measures were implemented at the onset of the pandemic and during the winter and spring of 2020/2021. In contrast, the summer months of both 2020 and 2021 were characterized by a gradual reopening of society and a relaxation of measures. Community mitigation measures were also periodically adjusted according to local infection rates, where high infection rates led to stricter measures [21]. In hospitals, guidelines mandated a prioritization of acute healthcare services [22], resulting in intermittent declines in elective admissions [23]. In instances where demand for intensive care exceeded the available capacity, guidelines prioritized patients with the highest expected benefit from treatment [24].

The municipalities were responsible for controlling the virus within their communities and nursing homes. To ensure a consistent response nationwide, the central government also issued nursing home-specific guidelines. The guidelines recommended that nursing homes should introduce measures to limit visitor access, implement enhanced cleaning and infection control protocols, and restrict hospital admissions for their residents. These steps were taken to increase hospital capacity and minimize the risk of in-hospital SARS-CoV-2 transmission [25, 26]. Screening measures for both staff and residents were also implemented. However, in the initial months of the pandemic, a shortage of SARS-CoV-2 tests resulted in a focus on testing symptomatic cases [27]. This limitation may have contributed to insufficient identification of asymptomatic individuals, potentially leading to the occurrence of clusters of infection in nursing homes at the onset of the pandemic. From May 2020, there was therefore a concerted effort to prioritize and enhance the use of SARS-CoV-2 tests in nursing homes, enabling a more comprehensive testing approach that also included asymptomatic cases [27].

Due to increased risk of severe disease and close interaction with health personnel, nursing home residents were given high priority when Norway received its initial doses of the vaccine in December 2020 [28]. By 21 February 2021, 91% of nursing home residents had received their first dose, and 82% had received two doses [29]. While most of the nursing home-specific measures remained in effect throughout our pandemic study period, there were some relaxations in February 2021 when most of the nursing home population was vaccinated [21]. A review of experiences at Norwegian nursing homes during the pandemic uncovered that most facilities successfully prevented initial entry of the virus, but that containing further transmission proved challenging once the virus was present within the facility. As a result, the majority of infections and COVID-19 fatalities among nursing home residents were concentrated within a select few nursing homes [30].

#### Data

To investigate mortality and hospitalization rates among nursing home residents during the pandemic, we used nationwide individual-level registry data from the Norwegian Emergency Preparedness Register, Beredt C19 [31], originating from the following registers: the Norwegian Population Register (demographic characteristics, including date of birth and gender); Individual-based Statistics for Nursing and Care Services, the Norwegian Patient Registry and the Norwegian Surveillance System for Communicable Diseases. The data sources were linked using a deidentified version of the personal identification number received upon birth or immigration to Norway.

### Study sample

Our pandemic cohort included all individuals who were permanent residents of nursing homes as of September 1st, 2019, totaling 30,052 people. They were fully exposed to the pandemic from its onset in March 2020. For comparison, we examined a pre-pandemic cohort consisting of 30,429 permanent nursing home residents on September 1st, 2017. Individuals in both cohorts were observed for up to 24 months or until death, from September 2017 to August 2019, and September 2019 to August 2021, respectively. This allowed us to analyze outcome trends 6 months before and 18 months after the pandemic onset. The pandemic might influence the composition of nursing home residents itself. For instance, heightened health concerns, changes in care availability and visitation restrictions might influence the entry of residents into nursing homes during the pandemic. In theory, it may also influence discharges from nursing homes, however, since we only considered long-term nursing home residents who required 24/7 supervision this is less likely. To avoid such compositional effects, we maintained a fixed cohort design by ensuring that the composition of nursing home residents in the analysis remained unchanged from their initial inclusion in September 2019 for the pandemic cohort, and September 2017 for the pre-pandemic cohort. No new admissions were accounted for, and no discharges were permitted, except in the event of death, thereby preserving the consistency of our sample for the duration of the 24-month follow-up period.

### Outcomes

Our primary outcomes of interest were all-cause mortality, all-cause inpatient hospitalization, and all-cause hospitalization before death. Inpatient hospitalizations were identified as all-cause overnight hospitalizations and included both acute and elective hospitalizations. Additionally, we studied the five most common cause-specific conditions for (inpatient) hospitalizations for nursing home residents (sickness in the circulatory system, respiratory system, digestive system, genitourinary system, injury, and other external causes) using the International Classification of Diseases, version 10 (ICD-10). We collapsed hospital admissions with less than two days between admission and readmission into a single hospitalization stay. In the analysis of cause-specific hospitalizations, we applied the main diagnosis of the first admission, as this was most likely the diagnosis that caused the nursing home to transfer the patient to the hospital.

We defined three indicators for our sample of nursing home residents: the Mortality Indicator is “1” in the resident’s month of death and “0” otherwise; the Hospitalization Indicator is “1” for any month with one or more overnight hospital stays, and “0” in other months; and the Hospitalization Prior to Death Indicator, which is “1” if a hospitalization occurred either in the month leading up to or during the month of death, and “0” otherwise.

### COVID-19 pandemic

Our intervention of interest was the COVID-19 pandemic. We treated the first lockdown in March 2020 as the start of the pandemic. To separate the effect of the pandemic from cyclical trends, we compared the change in mortality or hospitalization of nursing home residents in the pandemic cohort to outcome changes in the pre-pandemic cohort over the same period two years prior. To do this, we compared outcomes between the pandemic and pre-pandemic cohorts across a centered duration variable. The duration variable was centered in the last month before the pandemic started, February 2020, for the pandemic cohort, and February 2018, for the pre-pandemic cohort.

SARS-CoV-2 infection rates varied significantly in different regions in Norway [32]. The spread was higher in densely populated areas. We utilized this variation to explore differences in mortality and hospitalization rates between pandemic and pre-pandemic cohorts within areas with high and low community transmission rates. The measure of SARS-CoV-2 community transmission was defined as the total number of notified infections in each municipality in 2020 and 2021, divided by the total number of inhabitants in the same municipality. We linked each nursing home resident to the community transmission rates in the municipality where the nursing home was located. We then classified nursing home residents into low, medium, and high community transmission rates: those in the lowest 25% were placed in the low transmission group, those in the highest 25% in the high transmission group, and the rest in the medium group. Incidence rates ranged from 0 to 3.48 per 100 inhabitants in low, 3.49 to 8.74 in medium, and 8.74 to 14.3 in high transmission areas. See Additional file 1: Supplementary Fig. 1 for a map of these groups across Norway.

### Statistical analysis

We used the following event-study approach to examine the impact of the pandemic on nursing home residents’ mortality and hospitalization:

1 $${y}_{icm}= {\alpha }_{c}+{\gamma }_{m}+\sum_{k= -5,\ne -2}^{18}{{\pi }_{k}D}_{ck}+{\varepsilon }_{icm,}$$

where ${y}_{icm}$ represents an indicator for either mortality or hospitalization of individual *i* in cohort *c* in relative month *m.* The term ${\alpha }_{c}$ accounts for cohort-specific effects, controlling for unobservable effects that are constant to cohorts over time, and ${\gamma }_{m}$ is a relative month-specific effect that controls for seasonal effects that are common to both cohorts. ${D}_{ck}$ are relative month indicators equal to 1 for the pandemic cohort when month $m$ is month *k* ($k \in \left[\text{1,18}\right]$) of the pandemic or when there are $\left|k\right|$ months ($k \in \left[-\text{5,0}\right]$) before the pandemic arrives. The error term, ${\varepsilon }_{icm}$, is clustered at the individual level to account for within-individual correlation over time. The relative time index $k$ is set to 0 for February 2020 for the pandemic cohort and February 2018 for the pre-pandemic cohort.

By excluding *k* =  − 2, we designate this month (December 2019 for the pandemic cohort and December 2017 for the pre-pandemic cohort) as the reference month.^1^ Consequently, the relative time indicators’ (${\pi }_{k}$) measure the change in outcomes from the reference month (*k* =  − 2) to the specific months (*k* ranging from − 5 to − 3 and − 1 to 18) in the pandemic cohort relative to the change in outcomes for the pre-pandemic cohort over the corresponding months two years prior.^2^

These average comparisons would represent the pandemic’s impact if the change in outcomes for the pre-pandemic cohort represents how the outcomes would have changed for the pandemic cohort if the pandemic had not happened. Essentially, the changes in the pre-pandemic cohort outcomes are considered to reflect changes that would have happened anyway, without the pandemic. The assumption that the trends for both cohorts would have been equal without the pandemic, is known as the “parallel trends assumption,” which is the identifying assumption in event studies, as discussed in Cunningham’s book in chapter 9.4 [33].

To assess the parallel trend assumption, we tested whether there were significant differences in the outcomes in the pre-pandemic period (September 2019 to February 2020; *k* =  − 5, − 4, − 3…,0) for the pandemic cohort compared to the same seasonal period two years prior for the pre-pandemic cohort (September 2017 to February 2018; *k* =  − 5, − 4, − 3…,0).

### Difference-in-differences

Monthly mortality and hospitalization rates are likely to be influenced by short-term factors that could cause significant variation from one month to the next, which challenge the separation of any pandemic effects from natural fluctuations in monthly mortality. Therefore, we also estimate difference-in-differences (DD) models as well as a model where we grouped the months during the pandemic into seasons. In the former model, we compare the average pre-to-post pandemic percentage change for the pandemic cohort to the same period change for the pre-pandemic cohort two years prior.^3^ While in the latter model, we look at changes from the pre-pandemic period to individual seasons during the pandemic.^4^ The identifying assumption in the DD models is the same parallel trends assumption as in the event study, which posits that in the absence of the pandemic, the pandemic cohort and the pre-pandemic cohort would have followed the same trend over time in our outcome variables [33].

The event study and DD models capture the dynamic effects over time, allowing for an examination of mortality trends before and during the pandemic. In these models, there is a possibility of selective survival effects, where periods of increased mortality might lead to subsequent periods with relatively healthier population and consequently lower mortality. To systematically assess the net impact of such fluctuations on overall mortality, we utilized Kaplan–Meier Survival curves [34]. These curves will compare the survival probabilities of the pandemic and pre-pandemic cohorts over time. The comparison is statistically analyzed using the log-rank test to determine the significance of any differences observed between the two cohorts’ survival rates.

Additionally, we investigated whether COVID-19 mortality and hospitalization rates for nursing home residents differed according to different levels of community transmissions. More specifically, we compared outcomes for nursing home residents in municipalities with low, middle, and high community transmissions in the pandemic cohort, with outcomes for nursing home residents in the same municipalities in the pre-pandemic cohort. All regression estimates were presented for each month or season as a change in percentage points.

Statistical analyses were conducted using R version 4.2, and the modeling and clustering of standard deviations were performed using the “fixest” package with the vcov option [35], while the Kaplan–Meier survival curve [34] was calculated using the function ggsurvplot in the “survminer” package in R. The study adheres to the main principles of the STROBE guidelines for cohort studies [36].

## Results

### Descriptive statistics

In our study, we used the pre-pandemic cohort of nursing home residents as a baseline comparison group for those exposed to the pandemic. The fundamental assumption of our model is that, in the absence of the pandemic, health outcomes for the pandemic cohort would evolve in parallel to the pre-pandemic cohort. To assess the validity of this assumption, we compared both cohorts in terms of key demographic characteristics and level of community transmission before the pandemic. Our analysis revealed notable similarities between the groups: the average age was 85 years in both cohorts, and women constituted 70% of each cohort, as detailed in Table 1.

**Table 1:** Descriptive statistics on group characteristics for pre-pandemic and pandemic nursing home residents

	**Pre-pandemic cohort**	**Pandemic cohort**
**Sample characteristics**		
Residents, *N*	30,429	30,052
Age, mean (SD)	84.6 (8.9)	84.6 (8.8)
**Gender, *N* (%)**		
Females	21,372 (70.2)	20,970 (69.8)
Males	9057 (29.8)	9082 (30.2)
**Level of community transmission, *N* (%)**		
Low	7782 (25.6)	7409 (24.7)
Medium	15,059 (49.5)	15,225 (50.7)
High	7588 (24.9)	7418 (24.7)

The average health outcomes of our cohorts in the months before the pandemic (September 2017–February 2018 for the pre-pandemic cohort and September 2019–February 2020 for the pandemic cohort) were also similar (Table 2). The pre-pandemic and pandemic cohorts were thus similar in both demographics and health status, and it is therefore reasonable to expect that these cohorts would show similar trends in mortality and hospitalizations had the pandemic not occurred.

**Table 2:** Difference-in-differences of monthly mortality and healthcare utilization from before to after the onset of the pandemic

	**Pre-pandemic cohort**		**Pandemic cohort**			
**Health outcome, monthly %**	**Sep 2017–Feb 2018**	**Mar 2018–Aug 2019**	**Sep 2019–Feb 2020**	**Mar 2020–Aug 2021**	**Simple DD estimate**^**a**^	**ICD-10 codes:**
**All-cause mortality**	**3.53**	**3.29**	**3.49**	**3.32**	0.07	
**All-cause hospitalizations**	**3.34**	**2.68**	**3.22**	**2.29**	− 0.27***	
**Cause-specific hospitalizations (ICD-10 chapter)**						
Sickness in the circulatory system	**0.42**	**0.34**	**0.38**	**0.27**	− 0.03	I
Sickness in the respiratory system	**0.64**	**0.50**	**0.55**	**0.26**	− 0.15***	J
Sickness in the digestive system	**0.30**	**0.24**	**0.29**	**0.21**	− 0.02	K
Sickness in the genitourinary system	**0.32**	**0.29**	**0.34**	**0.26**	− 0.06**	N
Injury, poisoning, and certain other consequences of external causes	**0.66**	**0.53**	**0.67**	**0.58**	0.04	S
**Hospitalization prior to death**	**20.2**	**17.3**	**18.4**	**14.7**	− 0.80	
**Person-months:**	**167,557**	**336,310**	**165,159**	**331,555**		

^a^The estimate equals the difference between percentage change in monthly health outcome from before to during the pandemic for the pandemic cohort, and the corresponding percentage change in the pre-pandemic cohort, calculated as follows: ((monthly percentage outcome averaged over the pandemic period for the pandemic cohort) − (monthly percentage outcome averaged over the pre-pandemic period for the pandemic cohort) − ((monthly percentage outcome averaged over the post-period for the pre-pandemic cohort) − (monthly percentage outcome averaged over the pre-period period for the pre-pandemic cohort)). Standard errors (SEs) are clustered on individuals. Stars are used to denote the conventional levels of statistical significance, where * corresponds to a *p*-value of 0.1 or less, ** to a p-value of 0.05 or less, and *** to a *p*-value of 0.01 or less

The table shows the distribution of age, gender, and the SARS-CoV-2 community transmission level of the pre-pandemic and the pandemic cohort. The level of community transmission was calculated as the total number of SARS-CoV-2 infections in each municipality in 2020 and 2021, divided by the total number of inhabitants. Nursing home residents were divided into three groups, based on the community transmission in their municipality.

### Crude health outcomes for nursing home residents over time

**Fig. 1:**
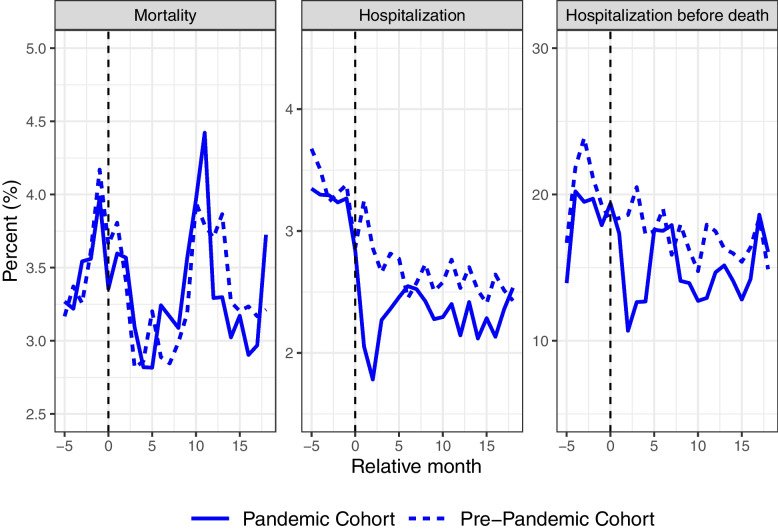
Crude monthly percent (%) of mortality and healthcare utilization among nursing home residents. Monthly percent (%) of nursing home residents who died, were hospitalized, and were hospitalized before death in the pandemic cohort (solid lines) and the pre-pandemic cohort (dashed lines). The *x*-axis refers to the relative month (*k*). The pre-pandemic cohort was measured 24 months earlier. The dotted vertical line (*k* = 0) refers to February 2018 for the pre-pandemic cohort, and February 2020, which is the month before the onset of the pandemic, for the pandemic cohort

In Fig. 1, we plotted the crude trends in mortality, hospitalization, and hospitalization before death in the pre-pandemic and pandemic cohorts. In the left panel, we observe that the trend in mortality for the pandemic cohort (solid line) from September 2019 (relative month (*k*) =  − 5) to February 2020 (k = 0) was similar to the outcome trend for the pre-pandemic cohort from September 2017 (*k* =  − 5) to February 2018 (*k* = 0) (dashed line).

After the pandemic arrived, the mortality trend in the pandemic cohort continued to follow the trend of the pre-pandemic cohort. In total, the monthly mortality rate for nursing home residents in the pandemic cohort slightly decreased from 3.49% in the pre-treatment period to 3.32% in the post-treatment period, corresponding to a total decrease of 0.17 percentage points (Table 2). For the pre-pandemic cohort, the mortality rate decreased from 3.53 to 3.29%, which corresponds to a 0.24 percentage points decrease. Hence, the difference in mortality from the pre- and post-treatment period between the pre-pandemic and the pandemic cohort was 0.07 percentage points (see the note in Table 2 for the formula behind the simple DD estimate) (Table 2). This increase was not statistically significant.

In contrast, there was a noticeable decrease of 0.27 percentage points in hospitalizations following the pandemic’s onset (Fig. 1, Table 2). This trend implies that nursing homes adhered to revised guidelines designed to minimize hospital admissions among their residents (see the “Background” section for further details on the guidelines). Furthermore, it is plausible that the lockdown mitigated the transmission of infectious diseases, thereby diminishing the demand for hospital-based care for individuals in nursing homes. Supporting evidence for this interpretation is presented in Table 2 and Additional file 1: Supplementary Fig. 2, which shows a consistent decline in hospitalization rates for respiratory diseases throughout the study period. There was also a statistically significant reduction in hospitalizations due to sickness in the genitourinary system conditions, and a non-significant reduction in hospitalizations due to sickness in the circulatory system (Additional file 1: Supplementary Fig. 2 and Table 2). A reduction in the hospitalization rate of nursing home residents before death was also observed (Fig. 1), suggesting an increase in end-of-life care conducted within nursing homes during the pandemic.

In the following section, we present the results from the event study. In contrast with the simple DD comparison in Table 2, which outlines average estimates before and after the onset of the pandemic, the event study estimates offer a more comprehensive insight into how the pandemic influenced the health of nursing home residents over time by displaying monthly variation in rates of (i) mortality, (ii) hospitalization, and (iii) hospitalization before death throughout the study period.

### Regression results

**Fig. 2:**
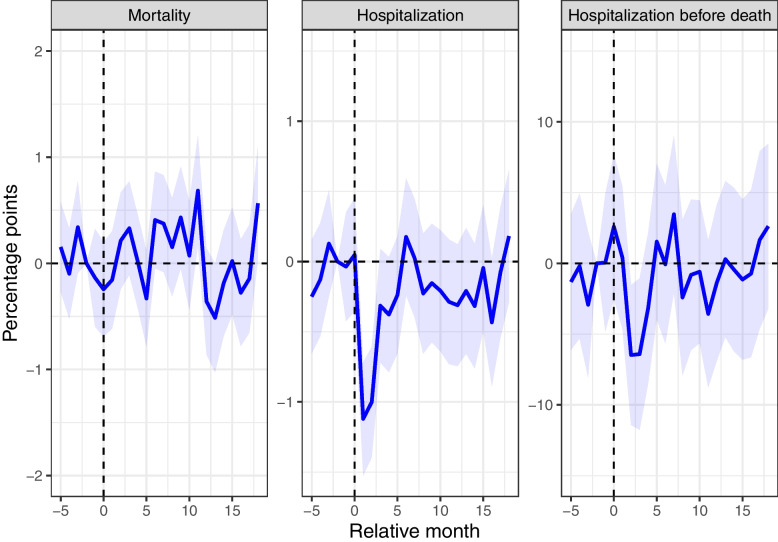
The impact of the pandemic on monthly mortality and healthcare utilization among nursing home residents. Estimated monthly difference (95% confidence interval (CI)) in mortality, hospitalizations, and hospitalizations before death for nursing home residents in the pandemic cohort and pre-pandemic cohort. The *x*-axis refers to the relative month (*k*). The pre-pandemic cohort was measured 24 months earlier. The dotted vertical line (*k* = 0) refers to February 2018 for the pre-pandemic cohort, and February 2020, which is the month before the onset of the pandemic, for the pandemic cohort

The results from the event regression specified in Eq. 1 are presented in Fig. 2. This figure illustrates that the mortality rates in both cohorts were comparable during the 6 months in the pre-treatment period (September 2017 to February 2018 (*k* =  − 5 to 0) in the pre-pandemic cohort and September 2019 to February 2020 (*k* =  − 5 to 0) in the pandemic cohort). The lack of statistically significant differences in mortality in this period supports the validity of the parallel trend assumption. After the onset of the pandemic, the line continued to fluctuate around the zero line until it became positive, albeit not statistically significant, in August 2020 (*k* = 6) when the second wave of the pandemic emerged. However, in the seasonal difference-in-differences estimates where all months during fall 2020 are collectively considered, a statistically significant increase in the average monthly mortality rate for the pandemic cohort of 0.31 percentage points is observed, representing a 9% increase from the pre-pandemic level (Table 3). The prior increase in the mortality rate leveled off during the winter 2020/2021. By spring 2021, the monthly mortality rates were 0.24 percentage points lower in the pandemic cohort, corresponding to a relative reduction in mortality of 7% in the pandemic cohort compared to the pre-pandemic cohort (Table 3).

**Table 3:** The impact of the pandemic on mortality and healthcare utilization among nursing home residents, by season

	**Pre %**	**Spring 2020**		**Summer 2020**		**Fall 2020**		**Winter 2020/2021**		**Spring 2021**		**Summer 2021**	
		**DD estimate (SE)**	**Rel. %**	**DD estimate (SE)**	**Rel. %**	**DD estimate (SE)**	**Rel. %**	**DD estimate (SE)**	**Rel. %**	**DD estimate (SE)**	**Rel. %**	**DD estimate (SE)**	**Rel. %**
**Health outcome**													
Mortality (all-cause)	3.49	0.11 (0.116)	3	0.02 (0.114)	0	0.31**(0.120)	9	0.13 (0.135)	4	− 0.24* (0.134)	− 7	0.03 (0.136)	1
Hospitalization (all-cause)	3.22	− 0.77*** (0.111)	− 24	− 0.11 (0.119)	− 3	− 0.07 (0.123)	− 2	− 0.22* (0.128)	− 7	− 0.16 (0.132)	− 5	− 0.07 (0.140)	− 2
Hospitalization before Mortality	18.40	− 3.60*** (1.295)	− 20	− 0.18(1.420)	− 1	0.41 (1.418)	2	− 1.56 (1.341)	− 8	− 0.12 (1.481)	− 1	1.49 (1.599)	8
**Hospitalizations (cause specific)**													
Sickness in circulatory system (I)	0.38	− 0.11*** (0.038)	− 30	0.02 (0.047)	5	− 0.03 (0.043)	− 7	− 0.00 (0.043)	0	− 0.03 (0.045)	− 8	− 0.03 (0.046)	− 9
Sickness in respiratory system (J)	0.55	− 0.27*** (0.046)	− 49	− 0.09** (0.047)	− 17	− 0.07 (0.048)	− 13	− 0.18 (0.052)	− 33	− 0.16 (0.054)	− 29	− 0.09 (0.059)	− 17
Sickness in digestive system (K)	0.29	− 0.04 (0.033)	− 12	− 0.01 (0.038)	− 5	0.01 (0.038)	3	− 0.06 (0.037)	− 19	0.02 (0.041)	6	− 0.02 (0.044)	− 6
Sickness in genitourinary system (N)	0.34	− 0.12*** (0.035)	− 35	− 0.03 (0.039)	− 9	− 0.06 (0.040)	− 17	− 0.03 (0.040)	− 8	− 0.06 (0.043)	− 18	− 0.03 (0.046)	− 10
Injury, poisoning, and certain other consequences of external causes(S)	0.67	− 0.02 (0.051)	− 3	0.04 (0.054)	6	0.05 (0.057)	8	0.06 (0.059)	9	0.11 (0.058)	17	0.02 (0.061)	3

Overall, the event study and seasonal difference-in-differences estimates of mortality fluctuate around zero. This phenomenon may be attributed to selective survival, wherein spikes in mortality during one period result in a relatively healthier population and fewer deaths in subsequent periods. To evaluate whether the later reductions in mortality offset the earlier increases, we present Kaplan–Meier survival curves in Additional File 1: Supplementary Fig. 3. Notably, the survival curves for the pandemic and pre-pandemic cohorts were remarkably similar, with a *p*-value from a log-rank test of 0.9.

The inpatient hospitalization rate declined by approximately 1 percentage point from February to March and April 2020 (*k* = 1, 2) (Fig. 2), before beginning to rise again in May 2020 (*k* = 3). On average, the reduction in the monthly inpatient hospitalization rate for the pandemic cohort in spring 2020 was 0.77 percentage points, representing a 24% decrease compared to the pre-pandemic level (Table 3). The reduction was primarily attributed to decreases in hospitalizations related to respiratory, genitourinary, and circulatory conditions, which decreased by roughly 49%, 35%, and 30%, respectively, compared to the pre-pandemic level. While there was a resurgence in hospitalization rates during the summer of 2020, the rates leveled off post-summer, maintaining a 2–7% reduction from the figures observed prior to the pandemic, as documented in Table 3.

The hospitalization rates before death exhibited a similar pattern to general hospitalization rates (Fig. 2). During the spring of 2020, there was a significant relative decrease in monthly hospitalization rates before death by 20% (Table 3). However, there were no significant differences in hospitalization rates before death between the pandemic cohort and the pre-pandemic cohort in the subsequent seasons.

### Subgroup analysis by community transmission levels

**Fig. 3:**
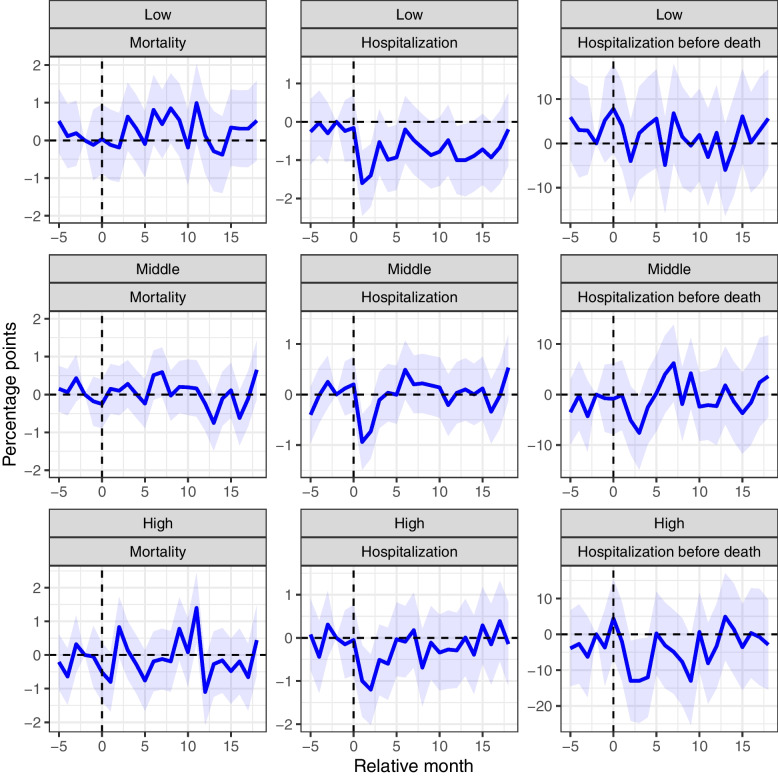
The impact of the pandemic on monthly mortality and healthcare utilization among nursing home residents in areas with low, middle, and high community transmissions. Estimated monthly difference (95% CI) in mortality, hospitalizations, and hospitalizations before death for nursing home residents in the pandemic cohort and pre-pandemic cohort by level of community transmission. The *x*-axis refers to relative month. The pre-pandemic cohort was measured 24 months earlier. The dotted vertical line (*k* = 0) refers to February 2018 for the pre-pandemic cohort, and February 2020, which is the month before the onset of the pandemic, for the pandemic cohort

The results from the event study model, estimated separately for nursing home residents in areas with low, medium, and high community transmission, are presented in Fig. 3 and Additional File 1: Supplementary Table 1 (for crude rates, see Additional File 1: Supplementary Fig. 4). The monthly mortality rate of nursing home residents in areas with relatively high community transmission rates spiked around the peak of the first and second waves of the pandemic (April/May 2020 (*k* = 2,3) and December 2020/January 2021 (*k* = 10, 11), respectively) (Fig. 3). However, as for the rest of the results on mortality for different levels of community transmission, the pandemic cohort and the pre-pandemic cohort were not statistically significantly different (Table A1). The probability of hospitalization significantly decreased for all nursing home residents during the first two months of the pandemic, regardless of the level of community transmission (Fig. 3). Interestingly, the estimated difference in hospitalization rates between the pandemic and pre-pandemic cohort in communities with high and middle transmission rates returned to the pre-pandemic level by summer 2020, while hospitalization rates for the pandemic cohort in communities with relatively low transmission rates stabilized at a significantly lower level (Fig. 3, Additional File 1: Supplementary Table 1). In areas with high levels of community transmission, nursing home residents were less likely to be hospitalized before death, particularly during spring and fall 2020 when the reduction was statistically significant (Additional File 1: Supplementary Table 1). The results for areas with low and medium levels of community transmission are less clear (Fig. 3, Additional File 1: Supplementary Table 1).

The first column shows the average monthly percent of pandemic residents that died/had at least one hospital admission/had at least one hospital admission prior to death in the 6 months before the pandemic (i.e., before March 2020), calculated separately for each outcome. Difference-in-difference estimates (DD estimates) quantify the change in health outcomes each season (March–May (spring); June–August (summer); September–November (fall); and December–February (winter)), measured as change in percentage points (DD estimates*100). Standard errors (SEs) are clustered on individuals. In addition to the presentation of results in absolute terms, relative differences in percent (Rel. %) are also presented, calculated by dividing the absolute estimate for each of the post-periods by the monthly average health outcome for the pandemic patients in the period prior to the pandemic (September 2019–February 2020, Pre %). Stars are used to denote the conventional levels of statistical significance, where * corresponds to a *p*-value of 0.1 or less, ** to a *p*-value of 0.05 or less, and *** to a *p*-value of 0.01 or less.

## Discussion

In this study, we have assessed the impact of the COVID-19 pandemic on mortality, hospitalization, and pre-death hospitalization rates among nursing home residents in Norway during the first 18 months of the pandemic. Notably, our findings reveal no significant overall increases in mortality rates, even in regions with high community transmission. However, certain periods showed noteworthy trends. For instance, a decline in mortality rates was observed in February 2021, when most of the nursing home population had received two doses of the COVID-19 vaccine. However, this decline may also be partly attributed to selective survival, as it occurred after a peak in mortality rates. Following a period of stabilization after April 2021, mortality rates rose again in August 2021, coinciding with the easing of community restrictions and the emergence of the Delta variant.

The absence of a clear overall increase in mortality during our study period may be interpreted as an indication that Norway’s proactive response, with early implementation of both national and nursing home-specific measures, effectively protected nursing home residents. The early enforcement of national restrictions was likely crucial in keeping infection rates lower than in most other European countries throughout the study period [37]. In addition, a comparison of infection rates in the general population with those in nursing home residents in 2020 shows that the infection rates among nursing home residents were lower (686 per 100,000 among nursing home residents versus 956 per 100,000 among all Norwegian inhabitants [28, 38]). This suggests that the nursing home-specific measures also effectively prevented the virus from entering these facilities.

Another important finding from our study is the substantial decrease in hospitalization rates among nursing home residents of 24% during the spring of 2020. This decline was primarily attributed to decreases in hospitalizations for respiratory diseases. Following this period, hospitalization rates remained stable, with no significant fluctuations observed after spring 2020.

During the spring of 2020, we also observed a notable 20% decrease in hospitalizations prior to death among nursing home residents. This trend was consistent across all areas but was most pronounced in regions with the highest community transmission rates. This suggests that nursing homes in regions with high community transmission rates were particularly conscientious in adhering to guidelines promoting on-site treatment for residents, aiming to mitigate hospital congestion.

While the high proportion of COVID-19 deaths in nursing homes has raised concerns, our study results suggest that this metric alone might not fully reflect the effectiveness of care homes in managing the pandemic. For instance, in Norway, nursing home fatalities constituted 60% of all COVID-19 deaths, one of the highest rates among countries within the Organization for Economic Co-operation and Development (OECD) [16]. Yet, our analysis indicates no significant increase in overall deaths among nursing home residents during the pandemic compared to the pre-pandemic period.

The study shows that Norway managed to reduce hospitalization rates of nursing home residents in the last months of life without experiencing increased mortality. A question is whether aging societies and communities can learn from the pandemic about how to effectively prevent the spread of contagious diseases to reduce hospitalization rates of nursing home residents and their associated costs. Within the evolving landscape of an aging population and the consequent challenges posed by healthcare capacity constraints, the implications of transitioning end-of-life care from hospitals to nursing homes emerge as an important topic for future research. That said, it is important to note that strict infection control measures are likely to have adverse effects on the quality of life of nursing home residents and their families. Restrictions on visitors and communal activities most likely led to social isolation and loneliness, which are associated with a wide range of adverse health effects, such as depression, anxiety, cognitive decline, and even premature death [3, 7]. Therefore, policymakers should consider unintended consequences and quality of life of nursing home residents and their families when implementing infection control measures in nursing homes.

Some strengths and weaknesses of this study are worth mentioning. First, a major strength of this study is the access to comprehensive administrative data covering the entire nursing home population in Norway and their health outcomes over time. This provided us with a unique opportunity to analyze and comprehend the potential impacts of the COVID-19 pandemic at the population level. Additionally, by comparing the health outcomes of nursing home residents exposed to the pandemic with nursing home residents two years earlier, we isolated the impact of the pandemic from secular trends in health outcomes by both age, duration of study, and period in a novel way. There are also some limitations. First, Norway has a relatively small population, resulting in fewer nursing home residents. This affects the precision of our estimates, necessitating caution when interpreting the results. Additionally, as a high-income country with extensive healthcare resources, the external validity of our findings to lower-income regions may be limited. Second, we were not able to disentangle the impacts of different community-wide and nursing home-specific preventative control measures. This is an inherent challenge in observational studies of pandemic response because many of these measures were implemented at the same time, making it difficult to identify their individual effects. Future pandemic responses should, where feasible, stagger the implementation of measures making it easier for future research to evaluate the impact of different measures. Third, we did not have access to comorbidity information in the nursing home registry. We therefore relied on prior hospitalization and mortality rates as proxies, which limits the analysis regarding health disparities between the pre-pandemic and the pandemic cohort. Fourth, our analysis does not account for variations in virus spread within municipalities due to the unavailability of specific location data for individual nursing homes. A fifth limitation of our study was the inability to follow cohorts through the Delta and Omicron waves due to data constraints. Throughout 2020 and 2021, the incidence rate of SARS-CoV-2 among nursing home residents in the most affected communities fluctuated between 8.75 and 14.30 cases per 100 people. However, during the Omicron surge from January to March 2022, Norway’s national incidence rate soared to 71.53 cases per 100 individuals [39]. This spike was largely driven by Omicron’s higher transmissibility and the easing of infection control measures in February 2022. However, since Omicron was associated with a lower risk of severe outcomes and the population had a high level of vaccination, mortality rates did not see a parallel rise. Nonetheless, a report by a report from the Norwegian Institute of Public Health indicates an increase in excess mortality in 2022, suggesting that this may be due in part to a backlog of deaths that were postponed by the stringent controls earlier in the pandemic [40].

## Conclusions

Our study contributes to the current understanding of how the COVID-19 pandemic has impacted the nursing home population. Contrary to expectations, our findings reveal no clear evidence of excess mortality in Norway during the pandemic, even in regions with relatively high rates of community transmission. These results suggest that the early implementation of nationwide and nursing home-specific infection control measures in Norway during the pandemic protected nursing home residents. Moreover, our research highlights the potential for infection prevention and control measures to reduce hospitalization rates among nursing home patients, without a corresponding rise in mortality. This insight is particularly valuable for policymakers looking to reduce expensive hospital stays. While there have been concerns regarding the disproportionate number of COVID-19 deaths in nursing homes, our study suggests that this metric may not accurately reflect the performance of nursing homes in preventing the spread of the virus. The disproportionate share of total pandemic deaths attributed to nursing home residents can largely be explained by nursing home residents being susceptible to infections.

### Supplementary Information

Additional file 1: Supplementary Fig. 1. “Classification of SARS-CoV-2 Community Transmission in Norway. This map illustrates the levels of community transmission across municipalities, categorized as low, medium, and high.” Supplementary Fig. 2. “Crude monthly percentages of cause-specific hospitalizations among nursing home residents.” Supplementary Fig. 3. “Kaplan–Meier Survival Curves for Pre-Pandemic and Pandemic Cohorts” Supplementary Fig. 4. “Crude monthly percentages of mortality and health care utilization among nursing home residents in areas with low, middle and high community transmissions.” Supplementary Table 1. “The impact of the pandemic on mortality and health care utilization among nursing home residents in areas with low, medium, and high community transmission, by season.”

## Data Availability

The findings of this study are based on data from the Norwegian Emergency Preparedness Register, which aggregates individual-level data across various Norwegian administrative databases, including those managed by the Norwegian Directorate of Public Health, Statistics Norway, the Norwegian Institute of Public Health, and the Norwegian Tax Administration. Due to restrictions, these data, accessed under a specific license for this study, are not openly available. Researchers wishing to use this data must submit a formal application, which is accessible at https://helsedata.no. This application should outline a comprehensive research proposal, detailing the project’s objectives and methodologies. Approval from the Regional Committees for Medical and Health Research Ethics is required, application for approval is available at rekportalen.no. The authors are willing to facilitate access to the data for interested researchers upon a justified request.

## Abbreviations

CI: Confidence interval
COVID-19: Coronavirus disease 2019
DD: Difference-in-differences
ICD-10: International Classification of Diseases, version 10.
OECD: The Organization for Economic Co-operation and Development
SD: Standard deviation
UK: United Kingdom
US: United States

## Footnotes

1 We need a reference month to compare *changes* in outcomes between the pandemic and pre-pandemic cohort. The reason we chose *k* =  − 2 rather than the more common *k* =  − 1 is because the period immediately preceding the pandemic is more likely to be influenced by anticipation and early effects of the incoming pandemic.

2 For example, the relative time coefficient for *k* = 1 measures the difference in outcomes of interest for the pandemic cohort in March 2020 compared to the baseline month (December 2019 (*k* = -2)) minus the change in outcomes for the pre-pandemic cohort during the same period two years prior (March 2018 (*k* = 1) compared to December 2017 (*k* =  − 2)). This coefficient helps isolate the impact of the pandemic by accounting for changes that would have occurred regardless of the pandemic.

3 This is calculated as follows: ((monthly percentage outcome averaged over the pandemic period for the pandemic cohort) − (monthly percentage outcome averaged over the pre-pandemic period for the pandemic cohort) − ((monthly percentage outcome averaged over the post-period for the pre-pandemic cohort) − (monthly percentage outcome averaged over the pre-period period for the pre-pandemic cohort)).

4 The model includes separate estimates for each season from March 2020 to February 2022 (March–May (spring); June–August (summer); September–November (fall); and December–February (winter)) and compared it to the 6 months before the pandemic. The estimating equation for this model is equal to Eq. (1), but where the relative time indicators are replaced by the season indicators.
