# Supplementary material for: All-cause mortality and hospital admissions for nursing home residents during the COVID-19 pandemic: a Norwegian register-based cohort study
Source: BMC Med. 2024 Aug 7;22:318. doi: 10.1186/s12916-024-03523-8 (PMC11304764; doi:10.1186/s12916-024-03523-8)
Supplement: Supplementary file 1 — Additional file 1: Supplementary Fig. 1. “Classification of SARS-CoV-2 Community Transmission in Norway. This map illustrates the levels of community transmission across municipalities, categorized as low, medium, and high.” Supplementary Fig. 2. “Crude monthly percentages of cause-specific hospitalizations among nursing home residents.” Supplementary Fig. 3. “Kaplan–Meier Survival Curves for Pre-Pandemic and Pandemic Cohorts” Supplementary Fig. 4. “Crude monthly percentages of mortality and health care utilization among nursing home residents in areas with low, middle and high community transmissions.” Supplementary Table 1. “The impact of the pandemic on mortality and health care utilization among nursing home residents in areas with low, medium, and high community transmission, by season.” [file 12916_2024_3523_MOESM1_ESM.docx]

# All-cause mortality and hospital admissions for nursing home residents during COVID-19

**Supplementary Figure 1: Classification of SARS-CoV-2 Community Transmission in Norway. This map illustrates the levels of community transmission across municipalities, categorized as low, medium, and high.**
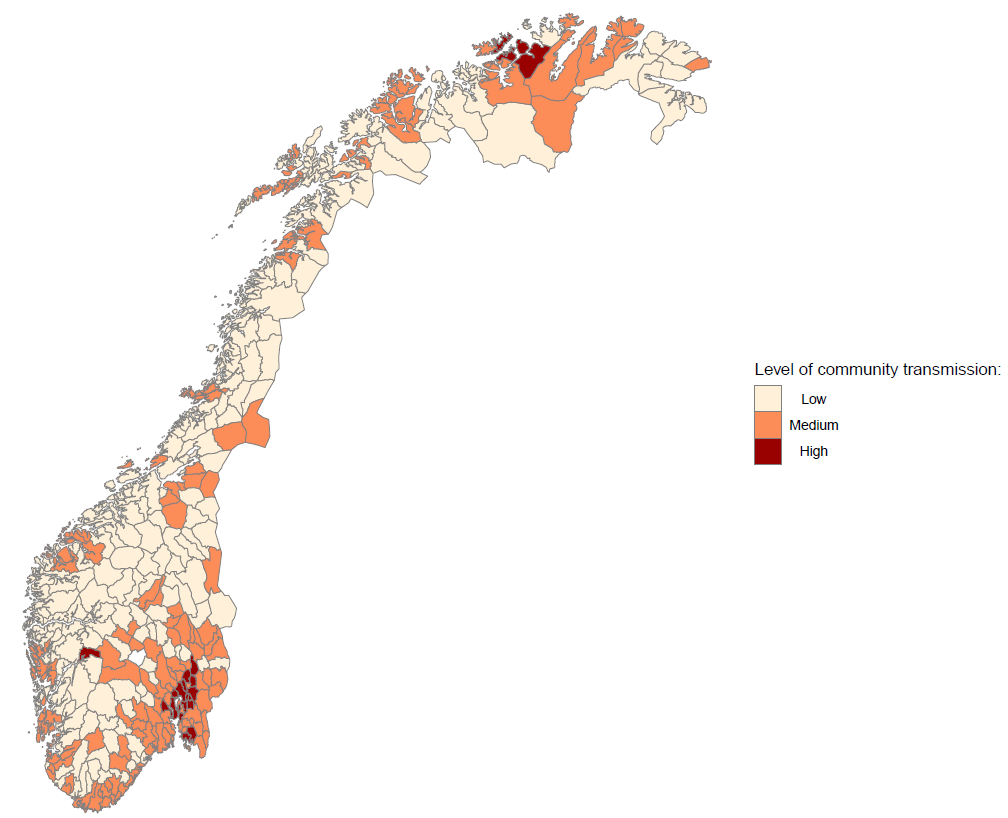
**Note:** The level of community transmission was calculated as the total number of SARS-CoV-2 infections in each municipality in 2020 and 2021, divided by the total number of inhabitants. Shape files used to create the map were obtained from (https://geonorge.no) and are licensed under Creative Commons BY 4.0 (CC 4.0).

**Supplementary Figure 2: Crude monthly percentages of cause-specific hospitalizations among nursing home residents**
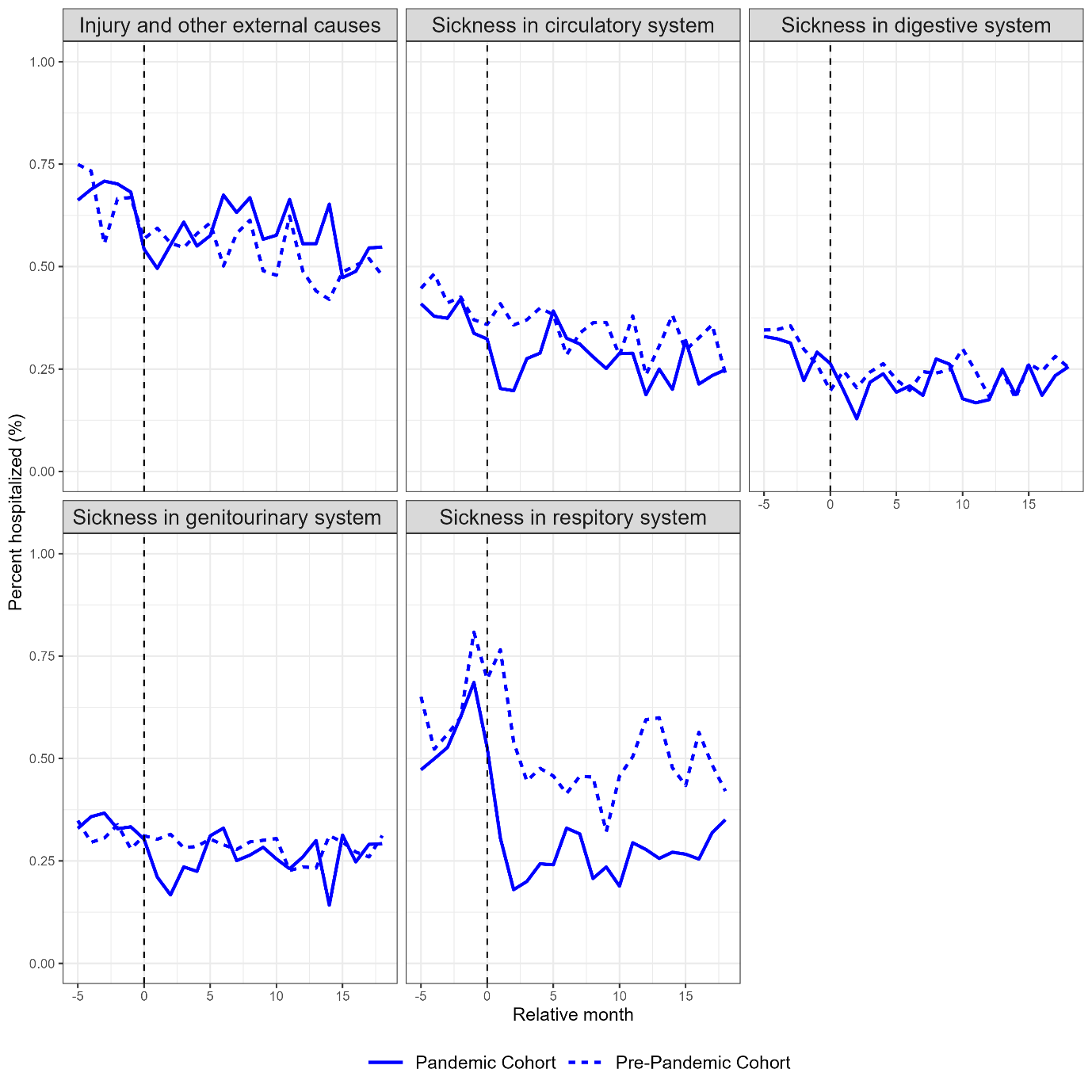


**Note:** Monthly percentage of nursing home residents who were hospitalized for different causes in the pandemic cohort (solid lines) and the pre-pandemic cohort (dashed lines). The x-axis refers to the calendar months where measurements were made for the pandemic cohort. The pre-pandemic cohort was measured 24 months earlier. The dotted vertical line (k = 0) refers to February 2018 for the pre-pandemic cohort, and February 2020, which is the month before the onset of the pandemic, for the pandemic cohort.

**Supplementary Figure 3: Kaplan-Meier Survival Curves for Pre-Pandemic and Pandemic Cohorts**


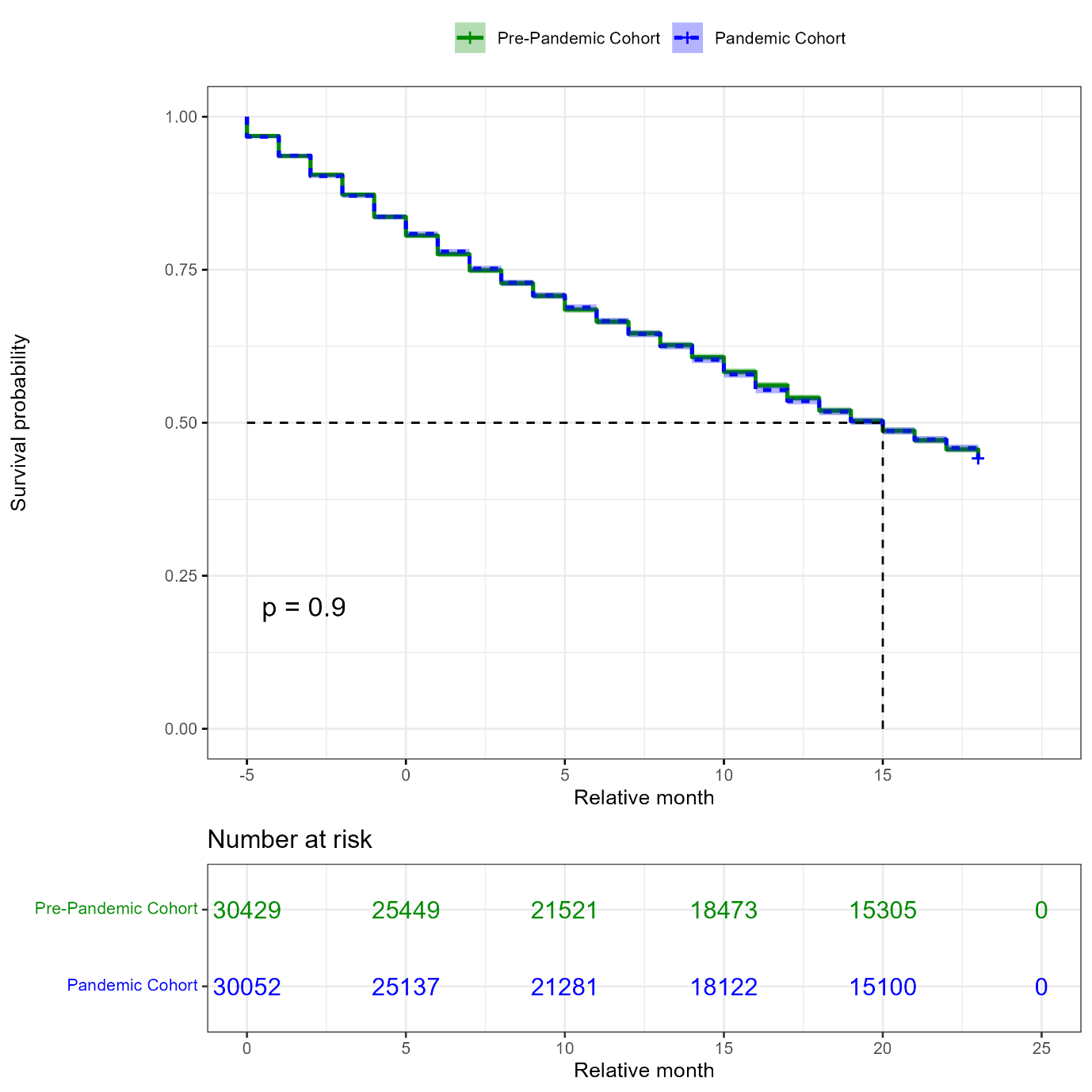


**Note:** Survival probability from start of follow-up (September 2019 and September 2017 (relative month (k) = 0) to end of follow-up (August 2021 and August 2018 (k=18)) were calculated separately for the pre-pandemic cohort (green lines) and the pandemic cohort (blue lines). The x-axis refers to the relative month. The pre-pandemic cohort was measured 24 months earlier. The dotted line shows the median survival, and the p-value are obtained from a log-rank test.

**Supplementary Figure 4: Crude monthly percentages of mortality and health care utilization among nursing home residents in areas with low, middle and high community transmissions.**
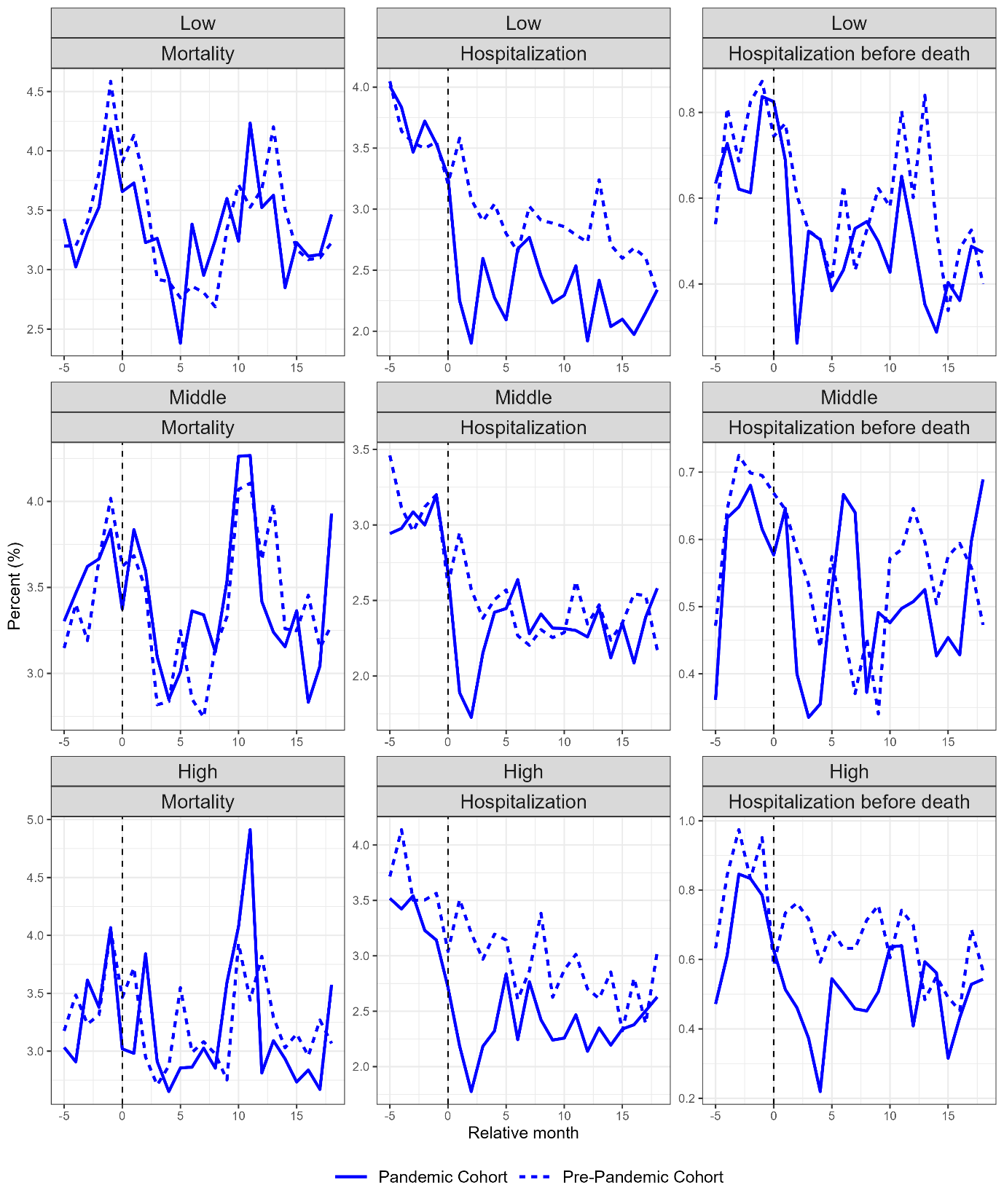


**Note:** Monthly percent (%) of nursing home residents who died, were hospitalized, and hospitalized before death in the pandemic cohort (solid lines) and the pre-pandemic cohort (dashed lines) in areas with low, middle and high community transmissions. The x-axis refers to the relative month (k). The pre-pandemic cohort was measured 24 months earlier. The dotted vertical line (k = 0) refers to February 2018 for the pre-pandemic cohort, and February 2020, which is the month before the onset of the pandemic, for the pandemic cohort.

**Supplementary Table 1: The impact of the pandemic on mortality and health care utilization among nursing home residents in areas with low, medium, and high community transmission, by season.**

|  | **Pre %** | **Spring 2020** | | **Summer 2020** | | **Fall 2020** | | **Winter 2020/21** | | **Spring 2021** | | **Summer 2021** | |
| --- | --- | --- | --- | --- | --- | --- | --- | --- | --- | --- | --- | --- | --- |
| Health outcome |  | DD estimate (SE) | Rel. % | DD estimate (SE) | Rel. % | DD estimate (SE) | Rel. % | DD estimate (SE) | Rel. % | DD estimate (SE) | Rel. % | DD estimate (SE) | Rel. % |
| **Mortality (all-cause)** |  |  |  |  |  |  |  |  |  |  |  |  |  |
| Low rates of community transmission | 3.52 | -0.07(0.236) | -2 | 0.18(0.228) | 5 | 0.46*(0.241) | 13 | 0.16(0.266) | 5 | -0.22(0.275) | -6 | 0.23(0.273) | 6 |
| Medium rates of community transmission | 3.55 | 0.15(0.165) | 4 | 0.06(0.163) | 2 | 0.22(0.172) | 6 | 0.00(0.194) | 0 | -0.31(0.191) | -9 | -0.08(0.195) | -2 |
| High rates of community transmissions | 3.33 | 0.23(0.226) | 7 | -0.23(0.227) | -7 | 0.33(0.237) | 10 | 0.34(0.268) | 10 | -0.12(0-256) | -3 | 0.04(0.265) | 1 |
| **Hospitalization (all-cause)** |  |  |  |  |  |  |  |  |  |  |  |  |  |
| Low rates of community transmission | 3.66 | -0.99***(0.235) | -27 | -0.57**(0.243) | -15 | -0.53**(0.254) | -14 | -0.58**(0.262) | -16 | -0.78***(0.276) | -21 | -0.45(0.287) | -12 |
| Medium rates of community transmission | 2.99 | -0.61***(0.151) | -20 | 0.15(0.166) | 5 | 0.19(0.169) | 7 | -0.03(0.176) | -1 | 0.05(0.181) | 2 | 0.03(0.193) | 1 |
| High rates of community transmissions | 3.27 | -0.87***(0.228) | -27 | -0.19(0.241) | -6 | -0.17(0.250) | -5 | -0.25(0.261) | -7 | 0.02(0.264) | 0 | 0.10(0.290) | 3 |
| **Hospitalization prior to death** |  |  |  |  |  |  |  |  |  |  |  |  |  |
| Low rates of community transmission | 20.14 | -3.53(2.554) | -18 | -2.81(2.879) | -14 | -1.61(2.906) | -8 | -3.59(2.817) | -18 | -5.58**(2.830) | -28 | -1.21(3.051) | -6 |
| Medium rates of community transmission | 16.45 | -2.14(1.778) | -13 | 2.30(1.964) | 14 | 4.55**(1.886) | 28 | -0.74(1.810) | -4 | 0.90(2.066) | 5 | 3.07(2.260) | 19 |
| High rates of community transmissions | 20.81 | -7.12**(2.791) | -34 | -2.71(2.919) | -13 | -6.31**(3.068) | -30 | -1.44(2.795) | -7 | 3.28(3.179) | 16 | 0.73(3.337) | 4 |

**Note:** The first column shows the average monthly percent of pandemic residents that died/had at least one hospital admission/had at least one hospital admission prior to death in the six months before the pandemic (i.e., before March 2020), calculated separately for each outcome. Difference-in-difference estimates (DD estimates) quantify the change in health outcomes each season (March-May (spring); June-August (summer); September-November (fall); and December-February (winter)), measured as change in percentage points (DD estimates*100). Standard errors (SEs) are clustered on individuals. In addition to the presentation of results in absolute terms, relative differences in percent (Rel. %) are also presented, calculated by dividing the absolute estimate for each of the post-periods by the monthly average health outcome for the pandemic patients in the period prior to the pandemic (September 2019-February 2020, Pre %). Stars are used to denote the conventional levels of statistical significance, where * corresponds to a p-value of 0.1 or less, ** to a p-value of 0.05 or less, and *** to a p-value of 0.01 or less
